# Fractalkine produced by Shiga toxin type 2-stimulated human glomerular endothelial cells modulates γδ T lymphocyte migration

**DOI:** 10.3389/fimmu.2026.1925614

**Published:** 2026-10-27

**Authors:** David Antonio Rosso, Micaela Rosato, Martín Ledesma, Fernando Daniel Gómez, Irene Angélica Keitelman, Florencia Sabbione, Analía Silvina Trevani, María Marta Amaral, Carolina Cristina Jancic

**Affiliations:** 1 Instituto de Medicina Experimental – CONICET – Academia Nacional de Medicina, Buenos Aires, Argentina; 2 Facultad de Ciencias Exactas y Naturales, Universidad de Buenos Aires, Buenos Aires, Argentina; 3 Laboratorio de Fisiopatogenia, Departamento de Ciencias Fisiológicas, Facultad de Ciencias Médicas, Universidad de Buenos Aires, Buenos Aires, Argentina; 4 Instituto de Fisiología y Biofísica Bernardo Houssay (IFIBIO Houssay), CONICET – Universidad de Buenos Aires, Buenos Aires, Argentina; 5 Departamento de Microbiología, Parasitología e Inmunología, Facultad de Medicina, Universidad de Buenos Aires, Buenos Aires, Argentina

**Keywords:** CX_3_CL1, fractalkine, CX_3_CR1, hemolytic uremic syndrome, Shiga toxin type 2, γδ T cells

## Abstract

**Introduction:**

γδ T cells play a critical role in tissue immunosurveillance through their capacity to sense cellular stress. However, their recruitment dynamics to peripheral organs such as kidney during Shiga toxin (Stx)–induced renal damage, remain undefined. Since the CX_3_CL1/CX_3_CR1 axis is vital for leukocyte homing into inflamed organs, we investigated its participation in mediating γδ T cell trafficking to the Stx–damaged kidney.

**Material:**

We integrated *in vitro* functional assays using Stx2a-stimulated human glomerular endothelial cells (HGEC) with *in silico* single-cell RNA sequencing (scRNA–seq) analysis of public human kidney injury datasets. CX_3_CL1/CX_3_CR1 expression was evaluated via qPCR, ELISA, and flow cytometry, while the functional recruitment γδ T cells recruitment was quantified using transwell migration assays.

**Results:**

*In vitro*, Stx2a significantly upregulates *CX3CL1* mRNA and protein secretion in HGEC. *In silico* profiling of acute kidney injury (AKI) tissues confirmed widespread *CX3CL1* upregulation in the damaged parenchyma and identified a distinct *TRGV9*+ cell cluster expressing *CX3CR1* transcripts. Contingency analysis showed that *TRGV9*+ cells were substantially more likely to express *CX3CR1* than other T/NKT cells. Importantly, flow cytometry analysis confirmed that γδ T cells express high levels of CX_3_CR1 protein. Finally, transwell assays demonstrated that both Stx2a-conditioned HGEC media and recombinant CX_3_CL1 potently induce γδ T cell migration, an effect that was completely abrogated upon disruption of the chemokine gradient.

**Discussion:**

Together, these findings suggest that CX_3_CL1 release by Stx2a-stimulated endothelial cells contributes to a chemotactic gradient that may mediate the targeted recruitment of circulating *CX3CR1*+ *TRGV9/TRDV2* cells to the injured kidney.

## 1 Introduction

Hemolytic Uremic Syndrome (HUS) is a late acute onset of symptoms that can appear after an initial intestinal infection with Shiga toxin (Stx)–producing *Escherichia coli* (STEC). In Argentina, HUS is highly prevalent; it is the most common cause of acute renal failure in children younger than five years old and the second cause of chronic renal failure. Furthermore, it is one of the leading causes of renal transplants in children and adolescents. Stx type 1 and type 2 (Stx1 and Stx2) produced by STEC O157:H7 and other strains are considered the main virulence factors associated with HUS that trigger kidney damage in patients. STEC strains expressing Stx2 are the most common pathogens responsible for severe cases of HUS in Argentina, and the Stx2a subtype causes the most serious illnesses (1). The clinical manifestations of HUS involve diarrhea, microangiopathic hemolytic anemia, thrombocytopenia, and variable degrees of kidney injury (2). During the hemorrhagic diarrhea stage, the disruption of the mucosa allows the Stx to enter the bloodstream and target organs expressing its receptors in their microvasculature, mainly in the kidney and central nervous system (3). Notably, the kidney is the most affected organ by Stx2a due to its high expression of Gb3 receptors and its role in blood filtration. Once in contact with endothelial cells, Stx2a activates them, inducing a pro–inflammatory state with an increase in adhesion molecules expression and cytokines and chemokines secretion, promoting the recruitment of leukocytes (4, 5). It has been reported that monocytes, NK cells, and neutrophils are involved in the pathogenesis of HUS (4, 6), nevertheless, the role of γδ T cells is poorly understood. γδ T cells represent 1–10% of the total T cell population in peripheral blood, and those expressing Vγ9Vδ2 TCR are the main circulating γδ T cells population in healthy humans (7). γδ T cells become activated by cytokines, molecules induced by stress, and microbial components (8). Once activated, γδ T cells release cytokines, such as IFN–γ and TNF–α; granzyme and perforin from cytolytic granules.

Zanchi et al. showed that the recruitment of NK cells and monocytes to kidneys was mediated by fractalkine (CX_3_CL1) (9). CX_3_CL1 is a member of the CX_3_C family of chemokines and participates in the interaction between inflammatory and endothelial cells (9). Interestingly, CX_3_CL1 acts as an adhesion molecule when expressed at the cell surface and as a soluble chemokine when its extracellular domain is cleaved (10). During immune responses, its expression could be increased by pro-inflammatory cytokines and microbial components (11). Recently, it has been proposed that CX_3_CL1 participates in the pathogenesis of HUS, since depletion of CX_3_CR1^+^ circulating mononuclear cells was observed during the acute stage of the syndrome, which correlates with the severity of renal failure, and the presence of monocytes and NK cells expressing CX_3_CR1 in the glomeruli (4). Interestingly, the pathogenic role of the CX_3_CL1–CX_3_CR1 axis has been described in both acute and chronic kidney diseases (12). Given that γδ T lymphocytes can infiltrate tissues (13), they may be actively recruited to the injured kidney via the expression of the CX_3_CL1 receptor: CX_3_CR1 (14). Previously, we demonstrated that soluble factors released by Stx2a-stimulated human glomerular endothelial cells (HGEC) induce the differentiation of γδ T cells towards a Th1-like profile. During this phenomenon, TNF–α plays a key role as its blockade abrogates the activation and differentiation of γδ T cells (15). Now, we aim to evaluate whether the conditioned medium derived from Stx2a–stimulated HGEC could also modulate γδ T cell motility. Furthermore, we wanted to analyze the contribution of CX_3_CL1 to this process, to expand the knowledge of this chemokine in modulating γδ T cell recruitment during the development of HUS.

## 2 Materials and methods

The experimental protocols performed were approved by the Biosafety and Research Review Board of Instituto de Medicina Experimental – CONICET – Academia Nacional de Medicina and the Ethical Committee of the Institutes of the Academia Nacional de Medicina and Universidad de Buenos Aires. The methods were carried out following the approved guidelines.

Detailed experimental procedures are provided in the **Supplementary Material**.

## 3 Results

### 3.1 Soluble factors released by human glomerular endothelial cells trigger γδ T cell migration

Previously, we demonstrated that soluble components released by Stx2a–stimulated HGEC modulate γδ T cell activation (15). Building on those findings, this work investigates whether these HGEC–derived soluble factors promote γδ T cell migration. To address this question, we first evaluated the capacity of HGEC conditioned media to induce γδ T cell migration by using a transwell system. Supernatants collected from Stx2a–stimulated HGEC significatively induced the migration of γδ T cells to the lower chamber of a transwell system (**Figure 1A**). This effect was consistently observed across the samples analyzed (**Figure 1B**). Of note, the concentration of Stx2a used in our experimental model did not induce γδ T cell activation (**Supplementary Figure 1**). It has been reported that soluble CX_3_CL1 is a strong chemoattractant for monocytes, T lymphocytes, and NK cells (16). Moreover, Zanchi et al. have reported that Stx2 induces CX_3_CL1 expression and it plays a key role in leukocyte–endothelial cell interactions (9). The authors proposed that it might contribute to renal microvascular dysfunction and thrombotic microangiopathy underlying HUS during enterohemorrhagic *Escherichia coli* O157:H7 infection (9). Furthermore, recent findings demonstrated a selective depletion of circulating CX_3_CR1^+^ mononuclear cells during the acute phase of the disease (4) further supporting the involvement of CX_3_CL1 in the pathogenesis of HUS. Interestingly, and in accordance with those previous reports (9), we observed an increase in *CX_3_CL1* mRNA in HGEC stimulated with Stx2a compared to the non-treated cells (**Figure 1C**). Remarkably, in concordance with the increase in *CX3CL1* mRNA level, we also observed an augmentation in the secretion of the chemokine by Stx2a–stimulated HGEC (**Figures 1D, E**).

**Figure 1:**
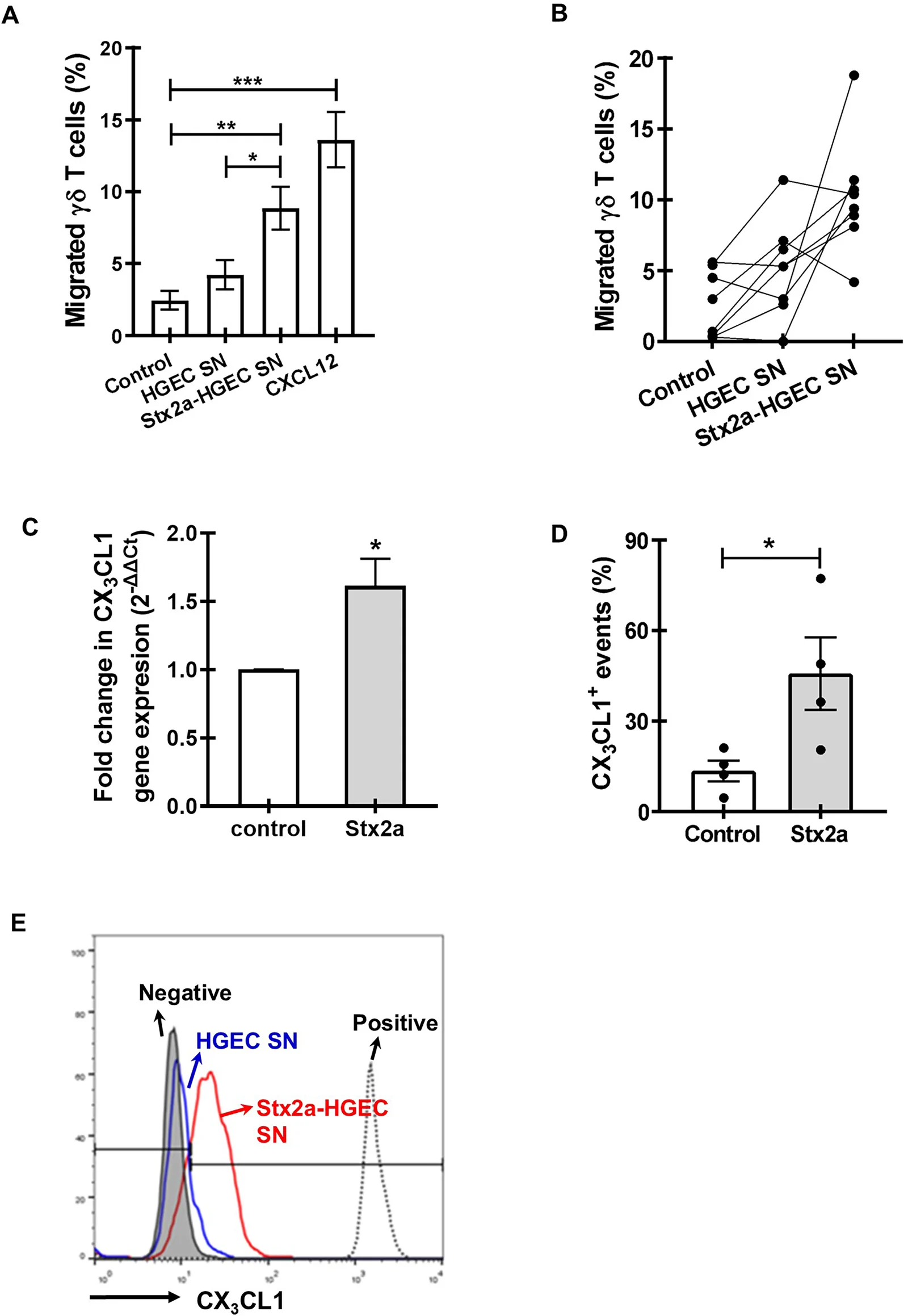
CX_3_CL1 production by Stx2a–stimulated HGEC. **(A, B)** γδ T cells were seeded in the upper compartment of a 96–transwell chamber, and conditioned medium from HGEC pre–treated (Stx2a–HGEC SN) or not (HGEC SN) with Stx2a (0.01 ng/ml, 24 h), were added in the lower compartment. Basal conditions were done using growth-arrested media instead of conditioned medium (Control). CXCL12: positive control. After 3 h of incubation at 37 °C, γδ T cells in the lower compartment (migrated cells) were quantified. A shows the mean ± SEM, n= 8 (biological replicates, where each n corresponds to a distinct γδ T cell donor). *p < 0.05, **p < 0.01 and ***p < 0.001, Kruskal Wallis test, with Dunn’s multiple comparisons test. **(B)** Shows paired individual samples from panel **(A)**, n= 8 (biological replicates, where each n corresponds to a distinct γδ T cell donor). Data in **(A)** were analyzed using an unpaired test to include the positive control, whereas **(B)** displays paired data from experiments evaluated in parallel using the same donors. **(C)** HGEC were treated with Stx2a (0.01 ng/ml). After 3 h of incubation, mRNA was obtained and processed to be analyzed by qPCR. Figure shows mean ± SEM of folds change in CX_3_CL1 gene expression, n= 3 (biological replicates). **p < 0.01, two-tailed paired *t*-test. **(D)** HGEC were treated or not (Control) with Stx2a (0.01 ng/ml). After 24 h of incubation, supernatants were recovered and the presence of CX_3_CL1 was analyzed by flow cytometry by employing LEGENDplex kit. Data are shown as the percentage of CX_3_CL1+ events in conditioned media from HGECs. Results are shown as mean ± SEM, n= 8 (biological replicates, where each n corresponds to a distinct γδ T cell donor). In **(A, B, D)** primary HGEC and the conditioned media were obtained from four individual (unpooled) donors and used across experiments. *p < 0.05, Mann Whitney test. **(E)** is a representative histogram of CX_3_CL1 obtained by flow cytometry. Basal: M199 supplemented with 10% FBS; Positive control: recombinant CX_3_CL1 (10 ng/ml) in M199.

### 3.2 Bioinformatics analysis of γδ T cells in the injured kidney

To investigate whether γδ T cell–associated transcriptional signatures are detectable in damaged human kidneys, we analyzed public scRNA-seq datasets from patients with acute kidney injury (AKI) and from a reference group samples (control donor) in the KPMP database. This focus aligns with experimental evidence supporting the view that AKI maintenance is driven by immune–mediated damage (17), suggesting a potential parallel with the pathophysiological processes we propose may be implicated in HUS. The analyzed T/NKT compartment comprised 4,129 cells from 32 independent specimens, including 3,525 cells from 12 AKI specimens and 604 cells from 20 control donor specimens. At the global level, AKI and control donor cells showed substantial overlaps in the Harmony–integrated transcriptional space, indicating no clear separation of the overall T/NKT compartment between conditions. Within this compartment, *TRGV9* expression was detected in 28/3,525 AKI cells (0.79%) and in 0/604 control donor cells, whereas *TRDV2* expression was detected in only 2/3,525 AKI cells (0.057%) and in 0/604 control donor cells (**Figures 2A, B**). Importantly, *TRGV9*–positive cells were distributed across 7 of 12 AKI specimens (58.3%), while none of the 20 control donor specimens contained *TRGV9*–positive cells. At the specimen level, detection of *TRGV9*–positive cells was associated with AKI status (Fisher’s exact test, *p* = 2.35×10^-4^). Given the very low frequency of positive cells, we interpreted these findings as an exploratory *TRGV9*/*TRDV2*–associated transcriptional signature rather than a definitive identification or expansion of a Vγ9Vδ2 T–cell population. To place this sparse KPMP signal in the context of γδ T–cell biology across tissues, we cross–referenced the findings with the CZ CELLxGENE platform (18). Analysis of annotated γδ T–cell populations showed the expected predominance of *TRDV2* and *TRGV9* in peripheral blood (7), whereas lymph node cells displayed a more balanced profile of *TRDV1*, *TRDV2*, and *TRGV9* (19); contrasting with their underrepresentation in mucosal barriers and solid tissues such as the liver, where *TRDV1* prevailed. In the kidney, although the γδ T–cell population was smaller in absolute number (approximately 1,700), *TRDV1, TRDV2*, and *TRGV9* showed high mean expression and were detected in a large fraction of the annotated γδ T–cell population, while TRDV3 expression remained marginal (**Figure 2C**). These complementary datasets therefore indicate that *TRGV9*/*TRDV2*–expressing γδ T cells can be detected in healthy human tissues when specifically resolved, while their detection within total kidney scRNA–seq datasets such as KPMP is sparse.

**Figure 2:**
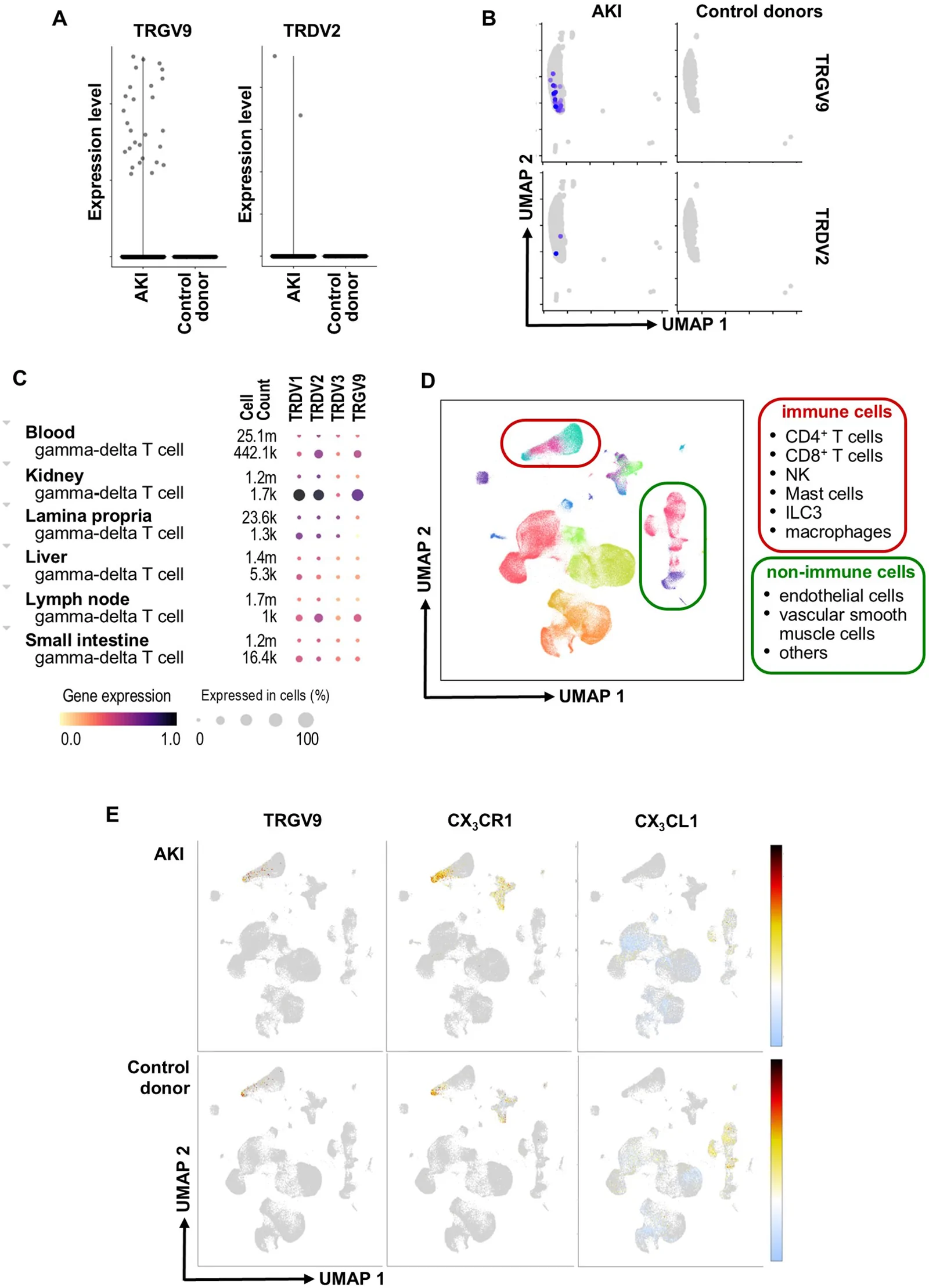
Bioinformatics analysis of γδ T cells in the kidney. **(A)** Violin plots of normalized RNA values. Each dot represents an individual cell expression level. **(B)** Uniform Manifold Approximation and Projection (UMAP) plots showing the transcriptional landscape of the T/NKT population and the spatial projection of *TRDV2* and *TRGV9* genes expression, obtained from public scRNA–seq data (KPMP data repository). Higher–expressing cells were blue colored. **(C)** Distribution of *TRDV1, 2, 3* and *TRGV9* transcripts in peripheral blood, kidney and lymph nodes in normal conditions, obtained from public scRNA–seq data CZ CELLxGENE. Dot color intensity represented the mean normalized expression level of each transcript within the cell population, while dot diameter reflected the proportion of cells expressing the corresponding gene. **(D)** UMAP of scRNA–seq data from kidney biopsies. Insets highlight detailed sub-clustering of lymphoid/immune cell lineages (red outline; e.g., T and NK cells) and vascular/stromal cell compartments (green outline; e.g., endothelial cells, pericytes, and fibroblasts). Cells are colored by cell type; see **Supplementary Table S1** for the complete color legend. **(E)** Plots comparing renal tissue from AKI patients and control donors. The color gradient indicates normalized and log–transformed gene expression levels, ranging from light blue/white (low/no expression) to dark red/black (high expression). In the KPMP T/NKT subset, 3,525 cells from 12 AKI specimens and 604 cells from 20 control donor specimens were analyzed; TRGV9 was detected in 28 AKI cells (distributed across 7 specimens) and TRDV2 in 2 AKI cells, while neither transcript was detected in control donor cells.

We next explored the potential involvement of the *CX3CL1*–*CX3CR1* axis in this setting. We observed that in public kidney scRNA-seq data, *CX3CR1* expression localized to the immune compartment, including cells expressing *TRGV9*, whereas *CX3CL1* expression mapped predominantly to non-immune renal cell populations and was visibly increased in AKI compared with reference tissue (**Figures 2D, E**). Within the AKI T/NKT compartment, 10 of 28 *TRGV9*–positive cells (35.7%) co–expressed *CX3CR1*, compared with 349 of 4,101 *TRGV9*–negative cells (8.5%), corresponding to a cell–level odds ratio of 5.96 (Fisher’s exact *p* = 6.86 × 10^-5^). Because cells from the same specimen are not independent biological replicates, we considered this cell–level analysis as exploratory and did not interpret it as patient-level inferential evidence. Overall, the transcriptomic data are consistent with a rare *TRGV9*–associated T–cell population in AKI with enriched *CX3CR1* expression, supporting the biological plausibility of responsiveness to *CX3CL1* within the injured kidney without establishing causality or paired Vγ9Vδ2 TCR identity.

### 3.3 Fractalkine modulates γδ T cell migration

To evaluate the response of γδ T cells to CX_3_CL1 *in vitro*; and test the *in silico* analysis, we first assessed the expression of its receptor, CX_3_CR1 by flow cytometry. **Figure 3** shows that γδ T lymphocytes express the highest levels of CX_3_CR1 among CD3^+^ lymphocytes (**Figure 3A**), and this expression was similar to that observed in CD3^-^ CD16^+^ cells (**Figure 3B**). As was previously described, we found that monocytes displayed the highest level of CX_3_CR1 compared with the lymphocytes populations analyzed (**Figure 3B**) (20). Then we studied the migration of γδ T cells in response to fractalkine. As **Figures 3C** shows, γδ T lymphocytes move towards CX_3_CL1 in the transwell system. To confirm the role of CX_3_CL1 in gd T cell migration induced by HGEC supernatants present in the lower chamber, these cells were placed in the upper chamber either in control medium or in medium containing a high concentration of recombinant CX_3_CL1 to disrupt the gradient. Interestingly, the presence of exogenous fractalkine in the upper compartment of the transwell abrogated γδ T cell migration (**Figures 3D, E**).

**Figure 3:**
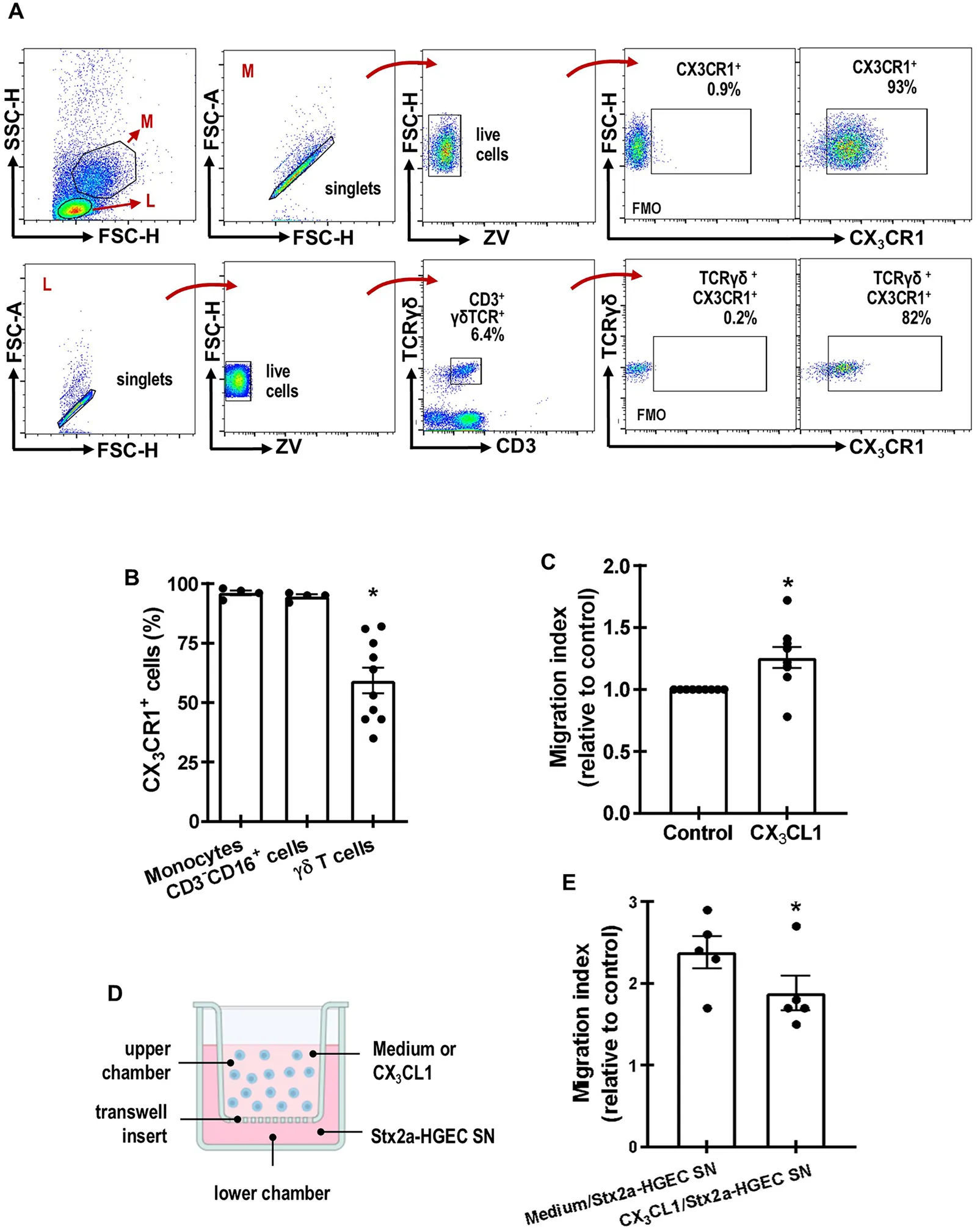
CX_3_CL1 promotes γδ T cell migration. **(A, B)** PBMC or purified γδ T cells were analyzed for the expression of CX_3_CL1 receptor by flow cytometry. **(A)** Representative dot–plots and histograms of CX_3_CR1 expression in PBMC subpopulations. *Gating strategy for CX_3_CR1 expression analysis*: Cells were first gated based on forward and side scatter (FSC/SSC) profiles to exclude debris. Monocytes (M) and lymphocytes (L) were identified based on their specific scatter properties, followed by doublet discrimination (FSC-A vs. FSC-H). Dead cells were subsequently excluded using Zombie Violet (ZV) viability dye. Within the monocyte gate, CX_3_CR1 expression was evaluated using fluorescence minus one (FMO) controls to set positive thresholds. Within the lymphocyte gate, cells were further sub-gated to define the CD3+ TCRγδ+ population prior to CX_3_CR1 assessment. Red arrows indicate the sequence in the gating strategy. **(B)** Mean ± SEM of CX_3_CR1 expression in different cell types, n= 4–10 (biological replicates, where each n corresponds to a distinct cell donor). *p < 0.05, Kruskal Wallis test. **(C)** γδ T cells were seeded in the upper compartment of a 96–transwell chamber, and CX_3_CL1 or medium alone (Control) were added in the lower compartment. After 3 h of incubation at 37 °C, γδ T cells in the lower compartmented (migrated cells) were quantified. Figure Shows the mean ± SEM, n= 9 (biological replicates, where each n corresponds to a distinct γδ T cell donor). *p < 0.05, Wilcoxon test. **(D)** Schematic representation of the protocol used in panel **(E)** designed using BioRender. **(E)** CX_3_CL1 or medium alone were added to the upper chamber together with γδ T lymphocytes. After 3 h of incubation at 37 °C, cells in the lower compartment (migrated cells) were counted. The panel shows the migration index relative to control in which medium was added in the lower and upper compartment of the transwells. Data are shown as the mean ± SEM. *p < 0.05, Wilcoxon test. Data represent n= 5 independent biological replicates, where each n corresponds to a distinct γδ T cell donor. In **(E)** primary HGEC-derived conditioned medium were obtained from four individual donors used across experiments.

## 4 Discussion

In HUS caused by STEC infection, the kidney is the most affected organ because glomerular endothelial cells express high levels of the Gb3 toxin receptor; in addition, the kidney has a higher exposure to Stx present in the blood after the infection, due to its blood filtering function. During HUS, the contact of endothelial cells with Stx favors the development of an inflammatory microenvironment, which allows the recruitment of immune cells (1–3). Altogether, this scenario allows the founding of local inflammation in which immune cells could contribute to renal damage. There is evidence for the role of innate immune cells, such as monocytes and NK cells, as key players in the pathogenesis of HUS (21). However, the involvement of γδ T cells in this process is not well established. Only a few reports have described their participation in bovines (22–24). Ruminants, mainly cattle, are the focus of these studies because they are the main reservoir of STEC serotypes associated with HUS (22). Understanding the infection process and persistence of bacteria in ruminants would go some way to preventing STEC infection in humans. Nevertheless, and since there are no specific treatments for HUS, it is extremely important to comprehend the intrinsic mechanisms of HUS development in humans, to manage the infection and the organ damage generated by Stx. In addition to the cytotoxicity induced by Stx, immune cells exacerbate the tissue damage originally generated by Stx. In this context, it has been reported that monocytes and NK cells are present in the kidney from HUS patients (4, 6), indicating that these cells are recruited to this organ. The chemokine involved in this process is fractalkine (9). Despite the presence of innate immune cells in the kidneys of HUS patients, there is no evidence of recruitment of γδ T cells in response to chemokines released by endothelial cells following Stx activation, even though γδ T lymphocytes are closely related to NK cells. Research in different kidney pathologies indicates that γδ T cells play a significant role in promoting immune–mediated renal injury (25, 26). According to this observation, a significant correlation between tubular cell injury and a peripheral decline in γδ T cells, but not in other T–cell subpopulations, was observed within the first 24 hours of AKI, supporting the role of γδ T cells in the very early phase of human AKI (27).

Previously, we demonstrated that γδ T lymphocytes play a role as pro–inflammatory cells in response to the microenvironment generated by Stx–stimulated renal endothelium. Indeed, we observed that γδ T cells increase IFN–γ and TNF–α production and CD107a expression, together with a decrease in intracellular perforin when they were incubated with Stx2a–treated HGEC–derived conditioned medium. Moreover, TNF–α was a key player during this process *in vitro* (15). To go further in the study of the role of γδ T cells in HUS development, in this work, we demonstrated that soluble factors secreted by human glomerular endothelial cells induce the migration of γδ T lymphocytes. Interestingly, consistent with that result, there was an increase in *CX3CL1* gene transcription and protein expression when renal endothelial cells were stimulated with Stx, in accordance with previous reports (9). It is noteworthy that the toxin concentration we used was set to achieve low mortality and moderate activation of HGEC. By this means, we could evaluate the behavior of γδ T cells in relation to endothelial cells, independently of the direct cytotoxicity induced by the toxin (15). Of mention, the concentration we used (0.01 ng/ml) is lower compared to previous reports where authors evaluate the effects of Stx on CX_3_CL1 production by endothelial cells treated with 3.5 ng/ml (9), a dose 350–fold greater than ours. Using low toxin concentration, we postulate that this condition recapitulates early events during the development of HUS, during which the toxin may be present at low doses in blood. Under this condition, Stx is unable to induce large endothelial cytotoxicity, but can help directly with the recruitment of immune cells to the affected endothelium. Supporting this hypothesis, we found that γδ T cells migrate towards CX_3_CL1 and, more interestingly, when we disrupted the chemotactic effect of this molecule in supernatants obtained from Stx2–stimulated HGECs, γδ T cell migration was reduced. We can speculate that among the factors secreted by renal endothelial cells, CX_3_CL1 could be a candidate to modulate γδ T cell recruitment. Certain limitations should be noted regarding the mechanistic link between Stx2a-HGEC supernatants and γδ T cell migration. Although qPCR and protein detection data confirm enhanced CX_3_CL1 secretion and migration assays prove its functional activity, we did not perform blocking experiments within the supernatant system. Consequently, the interpretation that CX_3_CL1 drives the supernatant-induced response is currently inferential, serving as a strong hypothesis for future mechanistic studies. Interestingly, the CX_3_CL1–CX_3_CR1 axis is an attractive therapeutic target due to its unique ligand/receptor pair, which avoids interactions with other partners, thereby reducing the potential for off–target effects (12). Given the lack of prior evidence for γδ T lymphocytes in the kidney during HUS, our goal was to extend exploratory human AKI observations from the findings obtained in the Stx2a–based experimental model, aiming to detect γδ T cells in the damaged renal tissue by analyzing public datasets from KMPM and CZ CELLxGENE Discovery. Interestingly, we observed a strong *TRGV9/TRDV2*–associated transcriptional signatures in the kidney, representing a subset typically predominant in peripheral blood, and renal enrichment was comparable to that observed in circulation. This is likely attributable to the dense vascular network of the renal tissue, which captures circulating cells and could increase during inflammation. Furthermore, given that this γδ T cell subset does not constitute the primary tissue–resident population in other steady–state organs, these findings could suggest a distinct, organ–specific microenvironment within the kidney that actively promotes the selective recruitment or retention of circulating Vγ9Vδ2 T cells. The transcriptomic profiles we obtained by dimensionality reduction analysis for *TRGV9*+, *CX3CR1* and *CX3CL1*, suggest that the localized release of *CX3CL1* by the injured renal parenchyma may establishes a chemotactic gradient, providing a potential receptor–ligand mechanism for the active recruitment of circulating γδ T cells to the damaged kidney. Of mention, the apparent lack of *TRGV9* and *TRDV2* expressions in the KPMP database compared to the robust detection in CZ CELLxGENE likely reflects technical differences rather than biological discrepancy. KPMP primarily utilizes sequencing on total tissue, which often lacks the sensitivity to detect rare immune populations and is prone to transcript dropout for TCR chains. In contrast, many datasets in CZ CELLxGENE employ whole–cell scRNA–seq, often following CD45+ enrichment, significantly enhancing the resolution for sparse circulating subsets like *TRGV9*/*TRDV2* cells. These findings suggest that while these cells are not abundant tissue–residents in healthy kidneys, their presence as a patrolling population can be uncovered through specialized cellular enrichment and whole–cell transcriptomics.

Together, our data may contribute to the understanding of early events that occur during the development of HUS. Of note, therapeutic decisions made at an early stage after STEC infection could prevent renal complications associated with blood dissemination of Stx, improving the quality of the patient’s life.

## Data Availability

The datasets analyzed for this study can be found in the KPMP: https://atlas.kpmp.org/, and CZ CELLxGENE DISCOVERY: https://cellxgene.cziscience.com/.
