# Supplementary material for: Fractalkine produced by Shiga toxin type 2-stimulated human glomerular endothelial cells modulates γδ T lymphocyte migration
Source: Front Immunol. 2026 Oct 27;17:1925614. doi: 10.3389/fimmu.2026.1925614 (PMC13747778; doi:10.3389/fimmu.2026.1925614)
Supplement: Supplementary file 1 [file DataSheet1.pdf]

Supplementary Data

1. Materials and methods

1.1 Reagents and antibodies

Ficoll-Hypaque was from GE Healthcare Bio-Sciences AB (Uppsala, Sweden). Anti-TCR γδ MicroBead kit was obtained from Miltenyi Biotec (Germany). RPMI 1640 culture media was from Invitrogen (Carlsbad, CA, USA). M199 media and endothelial cell growth supplement were from Sigma (MO, USA). Fetal bovine serum (FBS) was purchased from Internegocios (Buenos Aires, Argentina). Penicillin and streptomycin were from Gibco (USA). PerCP conjugated mouse antibody directed to CD3 (clone: OKT3), PE conjugated mouse antibody directed to CD16 (clone: 3G8), PE conjugated mouse antibody directed to CX_3CR1 (fractalkine receptor, clone: K0124E1), PE conjugated mouse antibody directed to TCR γδ (clone: B1), Zombie violet, and LEGENDplex to detect CX_3CL1 were from BioLegend (San Diego, CA, USA). Shiga toxin 2a (Stx2a) was from The Phoenix Lab, Tufts Medical Center (Boston, MA, USA). The human recombinant chemokine CXCL12/SDF–1 was purchased from PeproTech (DF, Mexico). SsoAdvanced Universal SYBR Green Supermix kit was purchased from BIO-RAD (CA, USA). Primers: GADPH Forward: 5’–GAGTCAACGGATTTGGTCGT–3’, GADPH Reverse: 5’–TTGATTTTGGAGGGATCTCG–3’, CX3CL1 Forward: 5–’TATCTCTGTCGTGGCTGCTC-3’ and CX3CL1 Reverse: 5’–CACAGACTCGTCCATTCCCC-3’ were from IDT (IA, USA).

1.2 Peripheral blood γδ T lymphocyte isolation

Peripheral blood mononuclear cells (PBMC) were obtained from heparinized human blood from healthy volunteers donors, who gave written informed consent, by centrifugation on the Ficoll–Hypaque gradient. Then, γδ T cells were purified by using magnetic microspheres covered with anti–TCR γδ antibodies, according to the manufacturer’s instructions. After purification, cells were resuspended in RPMI1640 supplemented with 10% heat-inactivated FBS, penicillin (100 U/ml), and streptomycin (100 μg/ml). Cells were analyzed by flow cytometry (FACSCalibur, Beckton Dickinson, CA, USA) to guarantee that γδ T cell purity was >98% and monocyte contamination <2% (doi:10.3389/fcimb.2021.765941). The total number of distinct donors used was 27.

1.3. Human glomerular endothelial cells culture

HGEC were isolated from healthy areas of the kidney fragments of patients undergoing nephrectomies as a consequence of segmental uropathies or tumors in one pole with normal creatinine. The procedure was performed at Hospital Nacional Alejandro Posadas, Buenos Aires, Argentina. Endothelial cells were isolated as was previously described (doi:10.3390/toxins11110648). Once obtained, cells were grown in M199 media supplemented with 20% FBS, 3.2 mM L-glutamine, 100 U/ml penicillin/streptomycin, and 25 μg/ml endothelial cell growth supplement. For growth-arrested conditions, a medium containing 10% FBS and without endothelial cell growth supplement was used. For the experiments, cells were used between passages 2 and 7. To treat HGEC, highly purified Stx2a was used. Lipopolysaccharide content on Stx2a was checked by the Limulus amebocyte lysate test (<10 pg/ml). The total

number of distinct donors used was 4. Cells from each donor were cultured and tested independently without pooling, with each donor representing an independent biological replicate.

1.4 Transwell assay

γδ T cells ($1.5x10^5$) in M199 supplemented with 1 % FBS were seeded in the upper compartment of a 96–transwell chamber with a polycarbonate filter of 5 µm pore size (Corning, MA, USA). Afterward, conditioned medium obtained from confluent monolayers of HGEC pre–treated or not with Stx2a (0.01 ng/ml, 24 h), were added in the lower chamber. Basal migration was determined by replacing the conditioned medium by growth-arrested media. After 3 h of incubation at 37°C, γδ T cells in the lower compartment (migrated cells) were recovered and quantified by flow cytometry.

1.5 qPCR

To measure the CX3CL1 mRNA produced by HGEC in response to Stx2a exposure (0.01 ng/ml), a qPCR was performed using the SsoAdvanced Universal SYBR Green Supermix kit. cDNA samples from HGEC were mixed with a solution containing the commercial supermix and the specific forward and reverse primers. Afterwards, the tube strips were placed in the CFX Connect Real–Time PCR System (BIO–RAD, CA, USA) thermocycler conigured to run 45 cycles of 10 s at 95ºC and 30 s at 58.5ºC.

The results obtained by qPCR were analyzed using the 2(–ΔΔCt) method, using GADPH as the reference gene. Each condition tested was done in triplicate.

1.6 CX_3CL1 detection

CX_3CL1 was measured in conditioned media obtained from HGEC cultures treated or not with Stx2a (0.01 ng/ml, 24 h), using the LEGENDplex liquid–phase chemokine detection kit (BioLegend, CA, USA) following the supplier's recommendations. Briefly, 25 µl of conditioned media or M199 medium (blank) was added to a V–bottom 96–well plate. Subsequently, 25 µl of assay buffer was added, followed by 25 µl of the capture beads (coated with specific antibodies for CX_3CL1). After 2 h of incubation on a shaker at room temperature, the plate was centrifuged, the supernatant was discarded, and a wash step was performed. Then, 25 µl of biotinylated detection antibody was added, the plate was incubated for 1 h at room temperature. Afterward, 25 µl of PE–conjugated streptavidin was added and incubated for an additional 30 min at room temperature. Finally, the wash steps were repeated, and samples were resuspended in 200 µl of wash buffer for acquisition on the flow cytometer. For the analysis, a forward scatter (FSC) versus side scatter (SSC) gate was first established to identify the population of capture beads. From this gate, the PE fluorescence intensity was analyzed, which corresponds to the presence of CX_3CL1 in the sample. As a positive control 10 ng/ml of recombinant CX_3CL1 was used.

1.7 Immunostaining and flow cytometry

PBMC were stained with Zombie Violet (ZV) viability dye, PerCP-conjugated anti-CD3, PE-conjugated anti-CX_3CR1, and FITC conjugated anti-TCRγδ. For flow cytometry analysis, cells were first gated based on forward and side scatter (FSC/SSC) profiles to exclude debris, followed by doublet discrimination (FSC-A vs. FSC-H). Dead cells were excluded using the ZV dye. From the viable singlet population, monocytes and lymphocytes were identified based on their specific scatter properties. Within the lymphocyte gate, cells were further sub-gated to define the CD3+ TCRγδ+ population prior to CX_3CR1 assessment. CX_3CR1 expression was evaluated on both

monocytes and the CD3+ TCRγδ+ subset, using fluorescence minus one (FMO) controls to establish positive thresholds. Acquisition was performed using FACSCalibur (BD Biosciences) and CyFlow Space (Sysmex Partec, Görlitz, Germany) cytometers. Data were analyzed using FlowJo software (v10.3 for Windows; TreeStar Inc., Ashland, OR, USA).

1.8 Bioinformatics

scRNA-seq data were sourced from the Kidney Precision Medicine Project (KPMP, https://atlas.kpmp.org/) integrated kidney atlas in .h5Seurat format and loaded into RStudio via SeuratDisk::LoadH5Seurat. The dataset included precomputed dimensionality–reduction embeddings, such as Harmony, PCA, and UMAP, along with cell–level metadata annotations. For this study, the data were filtered to focus on T/NKT immune populations using the curated cell type metadata field. Cells labeled as regulatory T cells (T-REG), cytotoxic T cells (T–CYT), natural killer T cells (NKT), and conventional T cells (T) were kept. A second filtering step selected only samples from acute kidney injury (AKI) and control donors, keeping cells with disease type annotated as AKI or Living Donor. Dimensionality reduction visualization used the Harmony embedding to depict the integrated transcriptional landscape of the T/NKT population. Expression of γδ T–cell–related genes *TRGV9* and *TRDV2* was analyzed within the T/NKT subset. These analyses aimed to determine whether γδ–like T–cell signatures differed between AKI and living donor kidney samples. Due to the sparse detection of T–cell receptor transcripts in scRNA–seq datasets and the predominance of zero-expression values, a binary co-expression strategy was adopted. Cells were classified as positive or negative for each marker according to the presence of detectable transcript expression (expression > 0). A 2 × 2 contingency table was generated comparing the frequencies of *CX3CR1*-positive and *CX3CR1*-negative cells among *TRGV9*–positive and *TRGV9*–negative populations. Statistical association between both markers was evaluated using Fisher's exact test. In parallel, Spearman correlation coefficients were calculated using normalized expression values to provide a complementary assessment of gene co-expression.

To characterize γδ T–cell receptor signatures across distinct tissue microenvironments, publicly available scRNA–seq datasets were explored using the CZ CELLxGENE Discover platform (https://cellxgene.cziscience.com/). Datasets containing annotated γδ T–cell populations derived from peripheral blood, kidney, and lymph node tissues were selected for comparative transcriptomic analysis. The analysis focused on the expression of canonical γδ T–cell receptor genes associated with tissue-resident and circulating γδ T cell subsets, including *TRDV1*, *TRDV2*, *TRDV3*, and *TRGV9*. Tissue–resident γδ T–cell populations were evaluated primarily by *TRDV1* and *TRDV3* expression, whereas circulating γδ T–cell populations were characterized by *TRDV2* and *TRGV9* expression, in accordance with previously described γδ T cell compartmentalization patterns. Cell populations corresponding to γδ T lymphocytes were identified using the original cell-type annotations provided by each dataset. Expression visualization and comparative analyses were performed using dot plot representations generated in the CELLxGENE interface. Comparative analyses were conducted descriptively across tissue types to evaluate differences in γδ T–cell receptor repertoire composition and transcriptional intensity within peripheral blood, kidney, and lymph node microenvironments.

For the KPMP analysis, independent biological specimens were identified using the SpecimenID metadata field. The final T/NKT subset included 32 independent specimens (12 AKI and 20 Living Donor) and 4,129 cells (3,525 AKI and 604 Living Donor). A cell was considered positive for a transcript when its normalized RNA expression value was greater than zero. Absolute positive cell counts and denominators were reported for *TRGV9* and *TRDV2*. To account for the

specimen structure when comparing *TRGV9* detection between disease groups, each specimen was classified according to the presence or absence of at least one *TRGV9*–positive cell, and a specimen–level Fisher's exact test was performed. *TRGV9*/*CX3CR1* co–expression was additionally summarized at the cell level using a 2 × 2 contingency table and Fisher's exact test; because cells are nested within specimens, this analysis was treated as exploratory enrichment and not as patient–level inferential evidence. Spearman correlation of normalized expression values was considered complementary only because of the high proportion of zero values characteristic of sparse TCR transcript detection.
For consistency across analyses, data from the KMPM database (originally annotated as "Living Donor") and CZ CELLxGENE discovery (annotated as "Healthy Donor") were unified and referred to as "control donor" throughout the study.

1.9 Statistical analysis

Statistical analysis was performed using GraphPad Prism v6.00 for Windows, GraphPad Software (La Jolla, CA, USA). Statistical significance was defined as $p<0.05$. The used tests are detailed in each figure.

Supplementary Figure

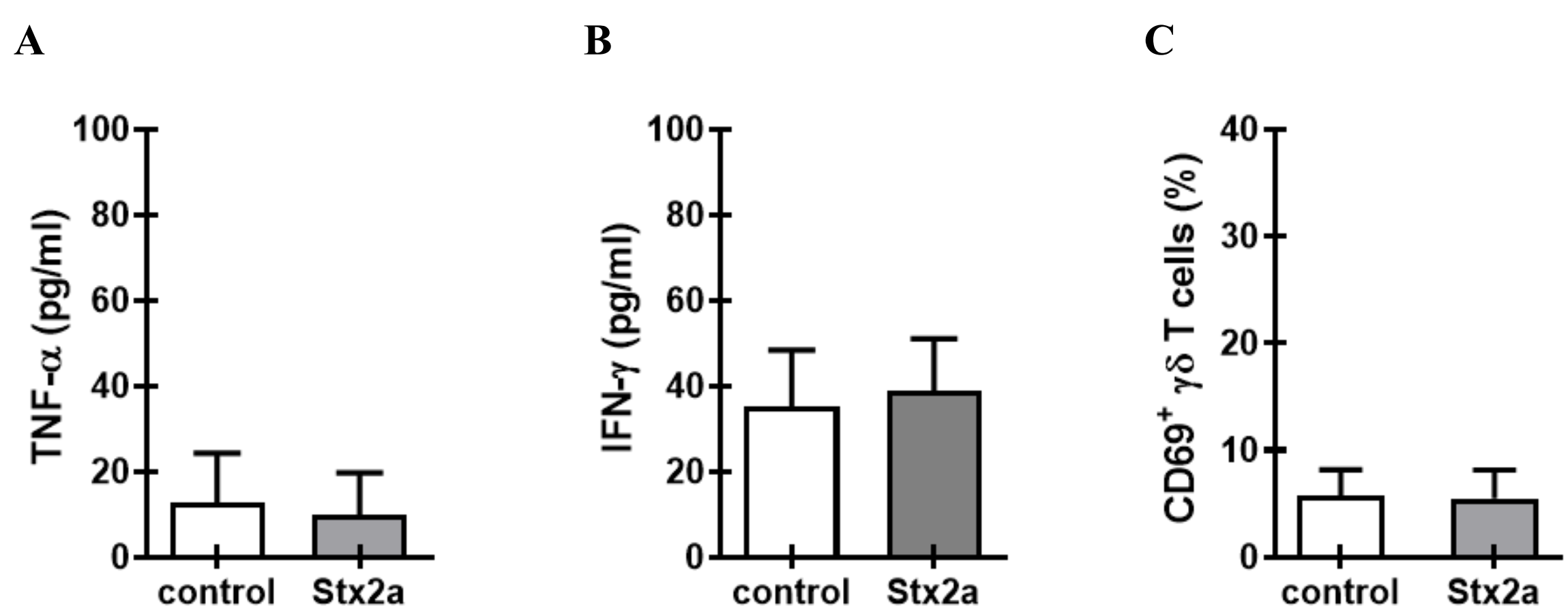

Supplementary Figure 1. Effect of Stx2a on γδ T cells. γδ T cells were treated or not with Stx2a (0.01 ng/ml, 24 h). Following incubation, supernatants and cells were collected. **A** TNF-α (n= 4) and **B** IFN-γ (n= 6) were measured by ELISA. Biological replicates. **C** CD69 expression in γδ T cells, analyzed by flow cytometry (n= 4), biological replicates. Results are shown as mean ± SEM.

Supplementary Table 1. Cells are colored according to the cell type metadata category. The legend indicates the identity of each cluster for each annotated phenotype.

B cell	
CD8-alpha-negative thymic conventional dendritic cell	
CD8-alpha-positive thymic conventional dendritic cell	
classical monocyte	
cycling macrophage	
effector memory CD8-positive, alpha-beta T cell	
effector memory CD8-positive, alpha-beta T cell, terminally differentiated	
endothelial cell	
epithelial cell of proximal tube	
group 3 innate lymphoid cell	
kidney collecting duct intercalated cell	
kidney collecting duct principal cell	
kidney connecting tubule epithelial cell	
kidney distal convoluted tubule epithelial cell	
kidney interstitial fibroblast	
kidney loop of Henle thick ascending limb epithelial cell	
kidney loop of Henle thin ascending limb epithelial cell	
kidney loop of Henle thin descending limb epithelial cell	
kidney resident macrophage	
macrophage	
mast cell	
migratory dendritic cell	
mucosal-associated invariant T cell	
naïve regulatory T cell	
naïve thymus-derived CD4-positive, alpha-beta T cell	
natural killer cell	
neural cell	
non-classical monocytes	
parietal epithelial cell	
plasma cell	
plasmacytoid dendritic cell	
podocyte	
renal interstitial pericyte	
